# Patient Onboarding and Engagement to Build a Digital Study After Enrollment in a Clinical Trial (TAILOR-PCI Digital Study): Intervention Study

**DOI:** 10.2196/34080

**Published:** 2022-06-13

**Authors:** Robert Avram, Derek So, Erin Iturriaga, Julia Byrne, Ryan Lennon, Vishakantha Murthy, Nancy Geller, Shaun Goodman, Charanjit Rihal, Yves Rosenberg, Kent Bailey, Michael Farkouh, Malcolm Bell, Charles Cagin, Ivan Chavez, Mohammad El-Hajjar, Wilson Ginete, Amir Lerman, Justin Levisay, Kevin Marzo, Tamim Nazif, Jeffrey Olgin, Naveen Pereira

**Affiliations:** 1 Department of Medicine University of California, San Francisco San Francisco, CA United States; 2 Department of Medicine University of Ottawa Heart Institute Ottawa, ON Canada; 3 Department of Medicine National Heart, Lung, and Blood Institute Bethesda, MD United States; 4 Department of Cardiovascular Medicine Mayo Clinic Rochester Rochester, MN United States; 5 Department of Medicine St. Michael's Hospital Toronto, ON Canada; 6 Department of Medicine University of Alberta Edmonton, AB Canada; 7 Department of Medicine University of Toronto Toronto, ON Canada; 8 Department of Medicine Mayo Clinic Health System La Crosse, WI United States; 9 Department of Medicine Minneapolis Heart Institute Minneapolis, MN United States; 10 Department of Medicine Albany Medical College Albany, NY United States; 11 Department of Medicine Essentia Institute of Rural Health Duluth, MN United States; 12 Department of Medicine Northshore University Health System Evanston, IL United States; 13 Department of Medicine Winthrop University Hospital Mineola, NY United States; 14 Department of Medicine Columbia University Medical Center New York, NY United States

**Keywords:** digital study, clinical trial, cardiology, smartphone, digital health, mobile health, clinical trial, mobile phone

## Abstract

**Background:**

The Tailored Antiplatelet Initiation to Lessen Outcomes Due to Decreased Clopidogrel Response After Percutaneous Coronary Intervention (TAILOR-PCI) Digital Study is a novel proof-of-concept study that evaluated the feasibility of extending the TAILOR-PCI randomized controlled trial (RCT) follow-up period by using a remote digital platform.

**Objective:**

The aim of this study is to describe patients’ onboarding, engagement, and results in a digital study after enrollment in an RCT.

**Methods:**

In this intervention study, previously enrolled TAILOR-PCI patients in the United States and Canada within 24 months of randomization were invited by letter to download the study app. Those who did not respond to the letter were contacted by phone to survey the reasons for nonparticipation. A direct-to-patient digital research platform (the Eureka Research Platform) was used to onboard patients, obtain consent, and administer activities in the digital study. The patients were asked to complete health-related surveys and digitally provide follow-up data. Our primary end points were the consent rate, the duration of participation, and the monthly activity completion rate in the digital study. The hypothesis being tested was formulated before data collection began.

**Results:**

After the parent trial was completed, letters were mailed to 907 eligible patients (representing 18.8% [907/4837] of total enrolled in the RCT) within 15.6 (SD 5.2) months of randomization across 24 sites. Among the 907 patients invited, 290 (32%) visited the study website and 110 (12.1%) consented—40.9% (45/110) after the letter, 33.6% (37/110) after the first phone call, and 25.5% (28/110) after the second call. Among the 47.4% (409/862) of patients who responded, 41.8% (171/409) declined to participate because of a lack of time, 31.2% (128/409) declined because of the lack of a smartphone, and 11.5% (47/409) declined because of difficulty understanding what was expected of them in the study. Patients who consented were older (aged 65.3 vs 62.5 years; *P=*.006) and had a lower prevalence of diabetes (19% vs 30%; *P*=.02) or tobacco use (6.4% vs 24.8%; *P<*.001). A greater proportion had bachelor’s degrees (47.2% vs 25.7%; *P<*.001) and were more computer literate (90.5% vs 62.3% of daily internet use; *P<*.001) than those who did not consent. The average completion rate of the 920 available monthly electronic visits was 64.9% (SD 7.6%); there was no decrease in this rate throughout the study duration.

**Conclusions:**

Extended follow-up after enrollment in an RCT by using a digital study was technically feasible but was limited because of the inability to contact most eligible patients or a lack of time or access to a smartphone. Among the enrolled patients, most completed the required electronic visits. Enhanced recruitment methods, such as the introduction of a digital study at the time of RCT consent, smartphone provision, and robust study support for onboarding, should be explored further.

**Trial Registration:**

Clinicaltrails.gov NCT01742117; https://clinicaltrials.gov/ct2/show/NCT01742117

## Introduction

### Background

The Tailored Antiplatelet Initiation to Lessen Outcomes Due to Decreased Clopidogrel Response After Percutaneous Coronary Intervention (TAILOR-PCI) was a large multicenter international randomized controlled trial (RCT) that compared point-of-care, genotype-guided P2Y12 inhibitor therapy to conventional clopidogrel therapy [1]. The initial follow-up duration of this trial was 1 year after the index percutaneous coronary intervention and randomization. Subsequently, the follow-up was extended to 2 years with 18- and 24-month study coordinator telephone visits. Notably, extending such follow-ups with in-person or telephone assessments of patients in large, multicenter RCTs such as the TAILOR-PCI RCT is expensive, time consuming, and complicated. The National Institutes of Health has recommended that RCTs be conducted in a pragmatic manner, including the use of digital technologies [2]. Recently, the importance of remote digital follow-up has been highlighted by the COVID-19 pandemic, during which many conventional RCTs requiring in-person recruitment and follow-up were stalled or suspended. Digital solutions to conducting RCTs provide increased convenience to both patients and enrolling sites and can potentially play a pivotal role in reducing costs and increasing accessibility to research. Given the near ubiquity of smartphones in many parts of the world and the use of mobile apps, remote RCT follow-up with regular data collection is now feasible. Whether digital technologies can be used to engage patients in a follow-up study once they are enrolled in an RCT is unknown.

The TAILOR-PCI Digital Study tested the feasibility of extending the original 1-year follow-up of the TAILOR-PCI RCT to a 2-year, remote follow-up using digital solutions and a low-contact approach (mailing letters and coordinator phone calls rather than clinic visits) to enrollment and engagement.

### Objectives

The objectives of this report are to describe our experience extending the follow-up and transitioning of the TAILOR-PCI pragmatic RCT to the TAILOR-PCI Digital Study after the main study had finished enrollment, with emphasis on patient onboarding, engagement, and results in the digital study.

## Methods

### Study Population

**Figure 1:**
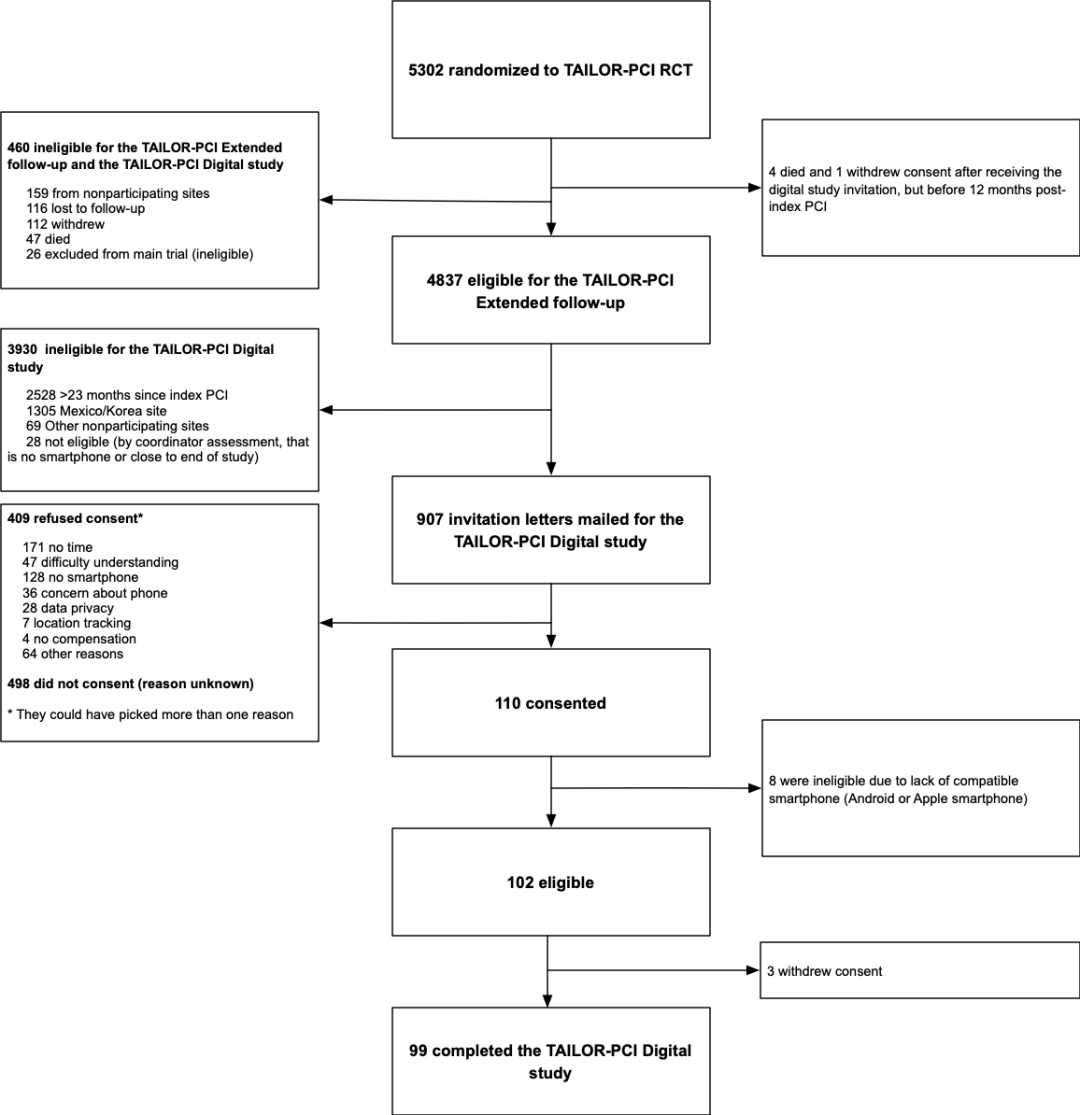
CONSORT (Consolidated Standards of Reporting Trials) diagram. PCI: percutaneous coronary intervention; RCT: randomized controlled trial; TAILOR-PCI: Tailored Antiplatelet Initiation to Lessen Outcomes Due to Decreased Clopidogrel Response After Percutaneous Coronary Intervention.

The parent TAILOR-PCI RCT (ClinicalTrials.gov NCT01742117) began enrolling patients on May 29, 2013, completed enrollment on October 31, 2018, and completed the final study follow-up a year later, with a study visit window open for up to 28 days thereafter. The TAILOR-PCI Digital Study tested the feasibility of extending RCT follow-up for up to 24 months in a subset of patients using a smartphone app designed for research. The design of the TAILOR-PCI Digital Study has been described previously [3]. Recruitment letters for the digital study were sent in February 2019. A digital study was built and conducted using the Eureka Research Platform, a direct-to-patient digital research platform [4]. TAILOR-PCI patients who enrolled from sites in the United States and Canada and were within 24 months of initial randomization and had an Apple or Android smartphone were eligible to participate (Figure 1). Of the original 34 US or Canadian TAILOR-PCI sites, 24 (71%) participated (3 declined and 7 did not have eligible patients).

### Recruitment

Recruitment was initiated by TAILOR-PCI site study coordinators who mailed letters to eligible patients and invited them to participate. Patients were instructed to visit the study website to learn more about the digital study, read and sign the consent form (if they chose to participate), and then receive an SMS text message with a link to download the study mobile app (Figure S1 in Multimedia Appendix 1). Embedded in the invitation letter was a unique patient code, a patient-specific *one-time access code* to establish 1:1 linkage with the TAILOR-PCI study ID, allowing synchronization of the data collected through Eureka and the RCT. Those who did not consent to the digital study after receiving the initial invitation letter were contacted via telephone by site coordinators and were asked whether they wanted additional information on the digital study or help with the smartphone app installation process and were encouraged to participate. Reasons for not participating were elicited (Multimedia Appendix 1), and data on the patients’ education level and computer literacy were also obtained.

### Oversight

The Mayo Clinic was the clinical coordinating center for all participating sites, and the University of California, San Francisco (UCSF) was the digital technology center. The UCSF developed the digital component of the study and provided technical support to the coordinating center throughout the study period. The Mayo Clinic conceived the study, received institutional review board (IRB) approval, and operationalized the implementation of the digital study, whereas the UCSF received IRB approval for the Eureka Research Platform as a digital coordinating center. Each participating eligible TAILOR-PCI site obtained local IRB approval for the study invitation material and for making patient contact. An independent National Heart, Lung, and Blood Institute–appointed Observational Study Monitoring Board was responsible for overseeing the conduct, safety, and data of the study.

### Ethics Approval

This study was approved by the Mayo Clinic IRB (number: 11-006837). The Eureka Platform used to conduct this study was approved by the UCSF IRB (number: 17-21879).

### Data Collection

At baseline, patients completed the following patient-reported outcomes (PROs) instruments on the mobile app: Duke Activity Score Index [5], Seattle Angina Questionnaire (SAQ) [6], and Modified Medical Research Council Dyspnea Scale [7] (Figure S1 in Multimedia Appendix 1). Patients also entered their medications with dosages using the Eureka medication tool [8]. These activities were repeated every month. The patients also completed a weekly 2-question angina diary. Anxiety scores were collected at baseline and every 6 months using the General Anxiety Disorder 7-item (GAD-7) questionnaire [9]. If monthly activities were not completed, patients received weekly automated SMS text messages and push notifications to remind them to complete the study activities.

### Aim of This Study

The main aim of the digital study was to determine the feasibility of transitioning a clinical trial to a digital study, and the aims were defined previously [3]. First, we described the proportion of patients who were invited to participate in the digital follow-up and consented. We further compared the patient characteristics of those who consented to participate with those who were eligible but did not consent. Second, among the consented patients, we measured the duration of participation in the study (duration in months between the first and last digital study activity completed by the patient). Third, we measured the proportion of enrolled patients who participated in at least 80% of the eligible digital visits (the total number of visits varied between 1 and 24 according to the date of enrollment in the main RCT). Additional outcomes that were measured were the proportion of consented patients who downloaded the Eureka app, average time until the study drop-off (described as skipping ≥1 month of activities and not re-engaging with the Eureka app despite Mayo Clinic digital study coordinator phone calls), digital visit completion rate (number of monthly digital visits completed over the number of visits available), attrition rate (1 minus the digital visit completion rate), and activity completion rate (number of activities completed over the number of activities available, stratified by weekly, monthly, and biyearly activities). Finally, as an exploratory aim, PROs collected in the digital study were described and stratified by the randomization arm in the parent trial.

### Data Analysis

The Duke Activity Score Index, Modified Medical Research Council Dyspnea Scale, SAQ, and GAD-7 were scored according to their respective instructions [5,7,9]. Continuous variables are presented as “mean (SD)” if approximately symmetrically distributed and as “median (IQR)” otherwise and were compared using the *t* test (2-tailed) or the Mann–Whitney *U* test as appropriate. Categorical variables were presented as “frequency (percentage)” and compared using either chi-square or Fisher exact tests, and 2-tailed *P* values <.05 were considered statistically significant, without further correction for multiple testing. Binary outcomes were reported with 95% CIs for the percentage using the Agresti-Coull method for interval estimation [10]. CIs for continuous variables were estimated using normal approximations for the mean, using transformations as needed. The digital visit participation rate and survey completion rates were calculated within participants to estimate a percentage and then summarized across individuals as continuous measures. Univariate logistic regression models were used to determine the effects of baseline characteristics (age, sex, etc) on the likelihood of consenting to the digital study. Significant variables in the univariate analysis (*P*≤.05) were included in this exploratory multivariable logistic regression model to identify the association between the different variables and the likelihood of consenting to the digital study. We present the activity results stratified by the randomization arm in the TAILOR-PCI RCT and grouped by bins of time since the index procedure. We assessed the survey results over time using a mixed model approach, allowing for random effects for intercept and slope within participants and modeled an overall intercept and slope as fixed effects, as well as with interaction between the randomized arm (genotype-guided vs conventional) and the slope. Data were analyzed using Python 3.5 and SAS (version 9.4; SAS Institute).

## Results

### Participation

Letters were mailed to 907 patients across 24 eligible sites in the United States and Canada (Figure 1), who had completed an average follow-up of 15.6 (SD 5.2; median 16.8, IQR 11.0-20.2) months since randomization in the parent RCT. In all, 2 sites mailed letters and had no patients enrolled in the digital study. These letters led to 31.9% (290/907 invited patients) study information webpage visits and 13.3% (121/907) registrations. A total of 12.1% (110/907) patients consented, among whom 92.7% (102/907) were eligible.

### Consent for the TAILOR-PCI Digital Study and Patient Characteristics

Of the 110 patients who consented, 45 (40.9%) did so after the invitation letter alone, whereas 37 (33.6%) and 28 (25.5%) consented after the first and second calls, respectively. The median time from randomization to invitation by letter of those who consented to the digital study as compared with those that did not consent was not different (median 17, IQR 5-23 vs median 17, IQR 4-24 months; *P=*.47). The mean age of the consented patients was 65.3 (SD 9.0) years versus 62.5 (SD 11.0) years for nonconsented patients (*P=*.006), and most of those who consented were male (91/110, 82.7% vs 594/797, 74.8%; *P=*.06). Comorbidities were similar among those who consented and those who did not, except cigarette use (7/110, 6.4% vs 198/797, 24.8%; *P<*.001) and diabetes (21/110, 19.1% vs 237/797, 29.7%; *P=*.02), which were less prevalent in the consented group (Table 1). A greater proportion of consenting patients had a bachelor’s degree or higher (75/106, 70.8% vs 233/620, 37.6%; *P<*.001). Among those who consented to participate in the education and computer literacy questionnaire, we observed a higher proportion of daily internet use (96/106, 90.5% vs 389/620, 62.7%; *P<*.001) and smartphone ownership (99/101, 98% vs 397/462, 86%; *P<*.001; Table 1).

**Table 1:** Baseline characteristics.

Variable				Consented patients (N=110)		Eligible patients (no consent) (N=797)		*P* value^a^	
**Hospital presentation randomization group, n (%)**									.62
	Stable coronary artery disease		33 (30)		214 (26.8)				
	Unstable angina or non-STEMI^b^		57 (51.8)		409 (51.3)				
	STEMI		20 (18.2)		174 (21.8)				
**Sex, n (%)**									.06
	Male		91 (82.7)		594 (74.5)				
	Female		19 (17.2)		203 (25.5)				
**Age (years)**									
	**At randomization**								.006
		Value, mean (SD)	65.3 (9.0)		62.5 (11.0)				
		Value, median (IQR)	65 (47-87)		62 (28-95)				
	**Men**								<.001
		Value, mean (SD)	65.6 (8.9)		61.2 (10.5)				
		Value, median (IQR)	65 (47-87)		61 (28-95)				
	**Women**								.38
		Value, mean (SD)	64.1 (9.5)		66.3 (11.7)				
		Value, median (IQR)	65 (47-81)		68 (36-95)				
**Ethnicity, n (%)**									.19
	White		99 (90)		657 (82.4)				
	Asian		3 (2.7)		27 (3.4)				
	African American		1 (0.9)		37 (4.6)				
	Hispanic or Latino		0 (0)		14 (1.7)				
	Other		7 (6.4)		62 (7.8)				
**Country, n (%)**									.61
	Canada		23 (20.9)		184 (23.1)				
	United States		87 (79.1)		613 (76.9)				
**BMI (kg/m^2^), n (%)**									.18
	<25		19 (17.3)		124 (15.6)				
	25-30		49 (44.5)		307 (38.5)				
	≥30		42 (38.2)		363 (45.5)				
Diabetes, n (%)				21 (19.1)		237 (29.7)		.02	
Hypertension, n (%)				74 (67.2)		554 (69.5)		.63	
Dyslipidemia, n (%)				77 (70)		540 (67.8)		.64	
Any history of heart failure, n (%)				2 (1.8)		28 (3.5)		.35	
Heart failure >2 weeks, n (%)				1 (0.9)		24 (3)		.21	
Estimated glomerular filtration rate (modification of diet in renal disease) <60, n (%)				14 (12.7)		91 (11.4)		.61	
Cigarette use, n (%)				7 (6.4)		198 (24.8)		<.001	
History of myocardial infarction (excluding index event), n (%)				14 (12.7)		136 (17.1)		.25	
Peripheral artery disease, n (%)				3 (2.7)		33 (4.1)		.48	
History of percutaneous coronary intervention, n (%)				27 (24.5)		220 (27.6)		.50	
History of coronary artery bypass grafting, n (%)				11 (10)		75 (9.4)		.84	
Stroke or transient ischemic attack, n (%)				2 (1.8)		26 (3.3)		.41	
Family history of coronary artery disease, n (%)				61 (55.4)		413 (51.8)		.48	
Chronic lung disease, n (%)				4 (3.6)		42 (5.3)		.46	
Currently on dialysis, n (%)				0 (0)		1 (0.1)		.71	
**Education and computer literacy form completed, n (%)**									<.001
	Completed the form		106 (96.4)		637 (79.9)				
	Did not complete the form		4 (3.6)		160 (20.1)				
	**Education level**								<.001
		Less than high school	2 (1.9)		39 (6.1)				
		High school graduate or some college	25 (23.5)		274 (43)				
		Associate or bachelor’s degree	50 (47.2)		164 (25.7)				
		Graduate or PhD	25 (23.6)		75 (11.7)				
		Prefer not to answer	4 (3.8)		85 (13.4)				
	**Frequency of internet use**								<.001
		Does not use	2 (1.9)		76 (11.8)				
		About daily	96 (90.5)		397 (62.3)				
		About once a week	2 (1.9)		46 (7.2)				
		Occasionally (less than once a week)	5 (4.7)		44 (6.9)				
		Do not know	0 (0)		2 (0)				
		Prefer not to answer	1 (0.9)		72 (11.3)				
	Has a computer or laptop		95 (93)		423 (88)		.17		
	Has a smartphone		99 (98)		404 (86)		<.001		
	Has a tablet		45 (49)		204 (46)		.63		
	Has a smart speaker		26 (31)		74 (19)		.02		
	Has downloaded app to phone		93 (94)		326 (81)		.006		

^a^Comparison of consented and nonconsented patients.

^b^STEMI: ST-elevation myocardial infarction.

### Nonparticipation in the Digital Study: Reasons and Predictors

Among the 409 patients surveyed during phone follow-up regarding reasons for nonparticipation in the digital study, the most common reasons reported (Table 2) were lack of time (171/409, 41.8%), lack of smartphone use (128/409, 31.3%), and difficulty in understanding what was expected of them in the study (47/409, 11.5%). Concerns about data privacy (28/409, 6.9%) and location tracking (7/409, 1.7%) were less frequent. Multivariable analysis revealed that older age, higher educational level, daily internet use, nonsmoking status, and nondiabetic status were significant independent predictors of consenting to the digital study (Table S1 in Multimedia Appendix 1). A sex–age interaction was observed, such that women aged 50 years were more likely than men at the same age to consent (odds ratio 1.55, 95% CI 0.58-4.16), but women aged ≥70 years were less likely to consent (odds ratio 0.46, 95% CI 0.24-0.87; interaction *P*=.03; Table S1 in Multimedia Appendix 1).

**Table 2:** Reasons given for declining participation in the digital follow-up.

Variable^a^	Overall participants (N=409), n (%)
No time	171 (41.8)
No smartphone	128 (31.3)
Difficulty in understanding	47 (11.5)
Concern about phone	36 (8.8)
Data privacy	28 (6.8)
No compensation	4 (1)
Location tracking	7 (1.7)
Other reasons	64 (15.6)

^a^Patients could choose more than 1 reason for not participating in digital follow-up.

### Engagement and Completion of Digital Visits and Activities

**Figure 2:**
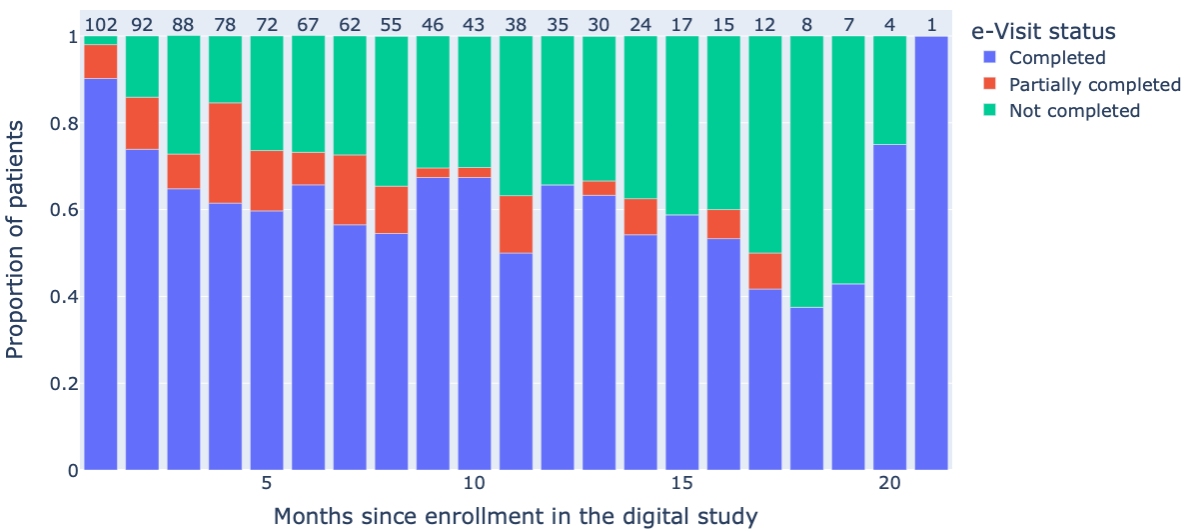
Tailored Antiplatelet Initiation to Lessen Outcomes Due to Decreased Clopidogrel Response After Percutaneous Coronary Intervention e-visit completion rate.

**Figure 3:**
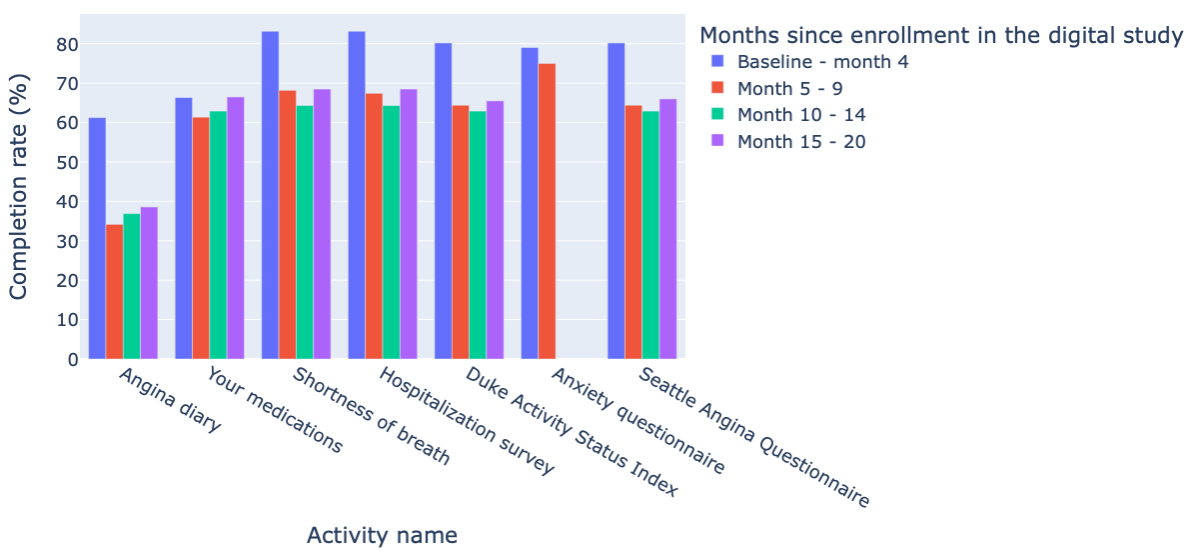
Tailored Antiplatelet Initiation to Lessen Outcomes Due to Decreased Clopidogrel Response After Percutaneous Coronary Intervention activities completion rates.

The median duration of follow-up for the digital study was 6.9 (IQR 3.0-12.3) months and patients participated for a median of 5.3 (IQR 2.2-10.9) months before dropping off. Until the date of the last follow-up, of the 102 consented and eligible participants, 65 (63.7%) remained engaged in the digital study, 61 (59.8%) patients completed ≥80%, and 41 (40.2%) completed <80% of all available digital visits (collection of surveys at one point in time). Among the 920 monthly digital visits made available to the patients, 577 (62.7%) were fully completed, 120 (13%) were partially completed, and 223 (24.2%) were skipped. The participation rate for the study e-Visits was constant throughout the course of the digital study (Figure 2), with patients completing 64.9% (SD 7.6%) of activities presented to them. A total of 55.48% (3525/6354) available activities were completed by patients, and this proportion increased to 67.06% (2443/3643) when the weekly angina diary was excluded (Figure 3). Out of the eligible patients, the completion rate of activities was 76.2% (78/102) between baseline and month 4, 62.1% (29/47) between month 5 and month 9, 59% (13/22) between month 10 and month 14, and 62.3% (9/14) between month 15 and month 20.

### PRO Findings

There were no differences among the randomization arms in any PROs (Figure S2 in Multimedia Appendix 1). There was a significant drop of >10 points in the SAQ disease perception (about 1.0 points less per 4 weeks; *P=*.02), but not in the other SAQ subdomains or in the overall SAQ score between the start of the study and the end of the digital study (Figure S2 and Table S2 in Multimedia Appendix 1). The patient Duke Activity Status Index trended lower throughout the study (approximately 0.4 points less per 4 weeks; *P=*.004; Figure S2 and Table S2 in Multimedia Appendix 1).

## Discussion

### Principal Findings

The TAILOR-PCI Digital Study was a proof-of-concept study that attempted to extend the follow-up of the main TAILOR-PCI RCT by using a digital platform at the end of the parent trial once enrollment was completed. The modest rate of participation at 12.1% (110/907) was limited by patients not visiting the home page after being mailed an invitation letter. Among those who responded to the invitation (171/409, 41.8%), perceived lack of time and lack of a smartphone were the most common reasons for declining participation.

There may be several reasons for the low participation rate in this proof-of-concept study. First, enrollment in the digital study began after completion of enrollment in the parent trial. The digital study was an *add-on* and was not integrated during initial recruitment in the parent RCT. Second, recruitment in the digital study used a low-touch approach with mailing letters and a limited number of phone call attempts (3 in total). Third, as email addresses were not collected in the parent trial, no email invitations were sent, and we relied on mailed paper letters for invitation to the digital study. There was a significant patient drop-off between the initial mail-in invitation to join the digital study and visits to the study webpage. One can speculate that the visit rate to the study webpage could have been increased by using email or SMS text message invitations and a higher-touch recruitment method, such as in-person enrollment during initial enrollment or subsequent follow-up in the parent trial.

Among those who consented (110/907, 12.1%), the engagement rate was excellent for a digital study that had minimal coordinator interaction, with 60% (66/110) of the patients completing 80% of the digital monthly visits. The digital study also demonstrated that once patients consented, the collection of a large amount (3525 digital PRO forms) and a wide variety of data, including a weekly angina diary, medications, the SAQ, and GAD-7, was feasible. This type of data can be used to phenotype patients enrolled in an RCT using digital technology.

### Reasons for High Digital Engagement and Retention Rates

The engagement and retention rates observed in our study among consented patients were higher than those reported in 12 previously described digital studies [11]. We did not observe an increase in the attrition rate during the duration of the study. This is in contrast to the median duration of participation of 5.5 days across several large-scale digital studies [11-15]. In these studies, only a fraction of patients contributed data from days 29 to 50 of recruitment [11-15]. For example, in a large asthma study, among 6470 consented patients, only 175 completed the required 6-month follow-up [13]. Several factors may have played a role in the higher engagement and retention rates observed in the TAILOR-PCI Digital Study. First, patients were already part of the TAILOR-PCI RCT and had maintained a 1-year clinical follow-up and therefore could represent patients who are more familiar with research protocols and more compliant with follow-up. Second, they experienced coronary artery disease, which is the clinical condition of interest studied in the digital study. Recruiting patients to a study that researches their medical conditions has previously been described as a factor of increased retention in digital studies [11,16]. Third, transparency in disclosing details of study participation, such as study duration, monthly time commitment, and data being collected, may have preselected motivated patients but could also have contributed to the lower than anticipated consent rate. Fourth, the observed lower attrition rate could also be owing to the several strategies used in the digital study to maximize engagement. Eureka has a robust automated messaging and reminder system that notifies patients when new activities are available or if they have not been completed.

### Challenges to Overcome and Possible Solutions to Implement Digital Technology in RCTs

Although the consent rate was lower than anticipated in our study, the 32% website visit rate observed in this study was higher than the 1% to 3% website visit rate reported in marketing campaigns that use emails to direct users to websites [17] or the 0.8% response rate for a large nationwide trial that used email invitations [18]. Successful recruitment for digital studies typically requires massive social media campaigns and a large number of invitations directed to a reasonable number of patients [11]. Moreover, experience with other studies on the Eureka platform and other digital studies suggests that higher-touch enrollment involving multiple phone call attempts or in-person enrollment during a study visit where technical concerns can be addressed at the time of digital enrollment are more effective [19], particularly in older populations such as those in TAILOR-PCI.

We identified several major challenges that should be addressed in future digital studies to increase consent rates. The digital study was designed and launched after enrollment in the main RCT was completed, thus requiring separate consent to be obtained months after the initial RCT enrollment. Therefore, we speculate that including a digital component at the inception of an RCT or at the time of initial consent may not only increase consent for digital studies but may also improve consent rates for RCTs by enabling easier follow-up. The lack of in-person visits with a study coordinator at the time of enrollment may have deterred the engagement of patients who had or perceived technical difficulties. For instance, among those eligible, 20% (1/5) of patients who did not participate in the digital study had never previously downloaded a smartphone app. A previous study demonstrated that in-clinic recruitment, as opposed to low-touch self-enrollment in a digital study, can increase both consent rate and engagement [11].

The lack of a smartphone was a major barrier to participating in the digital study, yet the prevalence of smartphone use, as reported by site study coordinators, was high at 75.8% (97/128) among those who cited it as a reason not to participate. This is comparable with the prevalence of smartphone use among those aged >65 years [20]. Although not directly explored in our study, patients may have lacked a compatible Android or Apple smartphone, may not have had a data plan, or may have been worried about the cost of data use. Strategies such as the provision of study-specific mobile devices or data plans to patients could enable easier implementation of digital technology in RCTs. Only 6.8% (28/409) of the patients who did not consent cited data privacy as a potential obstacle. We observed that data privacy concerns are often brought forward by IRBs and physicians as a potential barrier to digital studies, whereas patients themselves are less concerned [21]. Furthermore, the Eureka digital platform, which has engaged >400,000 study patients across 45 studies, is affiliated with an academic institution, the University of California San Francisco, which may inspire increased trust compared with commercial entities [22]. Finally, involving patients in the design and conduct of a digital study, which was not done for this digital study, could provide substantial value and lead to higher recruitment and engagement [23].

### Limitations

Our study has several limitations. Only a fraction of patients (110/907, 12.1%) from the main RCT consented for the digital study; therefore, our conclusions cannot be generalized to the parent trial cohort. Moreover, patients who consented to the digital study were not representative of those enrolled in the main RCT, as they were predominantly highly educated, healthier (did not smoke or have diabetes), of White ethnicity, and technologically literate. Although this disparity may affect RCTs, especially digital studies [24,25], specific efforts must be made to increase the recruitment of underrepresented minorities in digital studies. Strategies such as the inclusion of a smartphone with a data plan for eligible patients could reduce the accessibility barrier and possibly enroll more diverse populations. Although our study experienced higher engagement rates than other digital studies, dropout rates observed in digital studies were higher than those in standard RCTs (but not necessarily higher than follow-up registries after completion of the RCT follow-up); therefore, appropriate statistical considerations need to be given, including novel trial designs specifically applicable to mobile health studies. Finally, because of the lower than anticipated consent rate, we were able to obtain clinical information digitally only from a small subset of patients enrolled in the main RCT; therefore, our clinical findings are descriptive and hypothesis-generating.

### Conclusions

Extended follow-up of the TAILOR-PCI RCT using a digital platform was technically feasible; however, enrollment and consent rates in this study population were significantly limited. Once enrolled in the digital study, engagement was initially high, but the digital activity completion rate was modest. The reasons for low enrollment and modest activity completion rate by patients using this digital technology deserve further exploration.

## Appendix

Multimedia Appendix 1 Digital study.

## Abbreviations

GAD-7: General Anxiety Disorder 7-item
IRB: institutional review board
PRO: patient-reported outcome
RCT: randomized controlled trial
SAQ: Seattle Angina Questionnaire
TAILOR-PCI: Tailored Antiplatelet Initiation to Lessen Outcomes Due to Decreased Clopidogrel Response After Percutaneous Coronary Intervention
UCSF: University of California, San Francisco
