# Supplementary material for: Patient Onboarding and Engagement to Build a Digital Study After Enrollment in a Clinical Trial (TAILOR-PCI Digital Study): Intervention Study
Source: JMIR Form Res. 2022 Jun 13;6(6):e34080. doi: 10.2196/34080 (PMC9237778; doi:10.2196/34080)
Supplement: Multimedia Appendix 1 [file formative_v6i6e34080_app1.docx]

**Multimedia Appendix 1**

**Figure S1. TAILOR-PCI Digital Registry Overview**


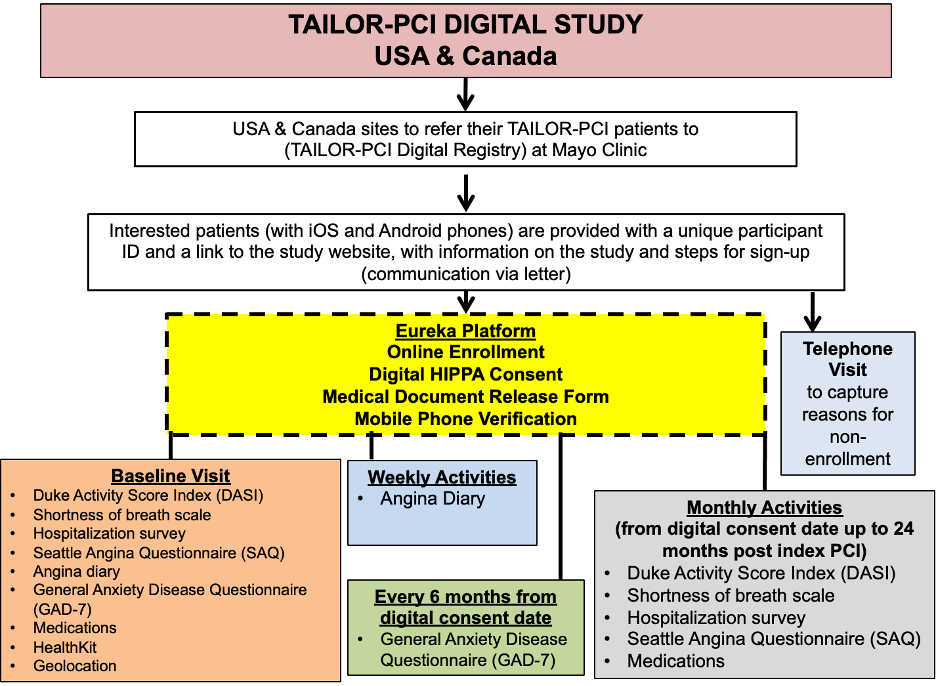


#### **Figure S2. Survey Answers**

#### Angina Diary


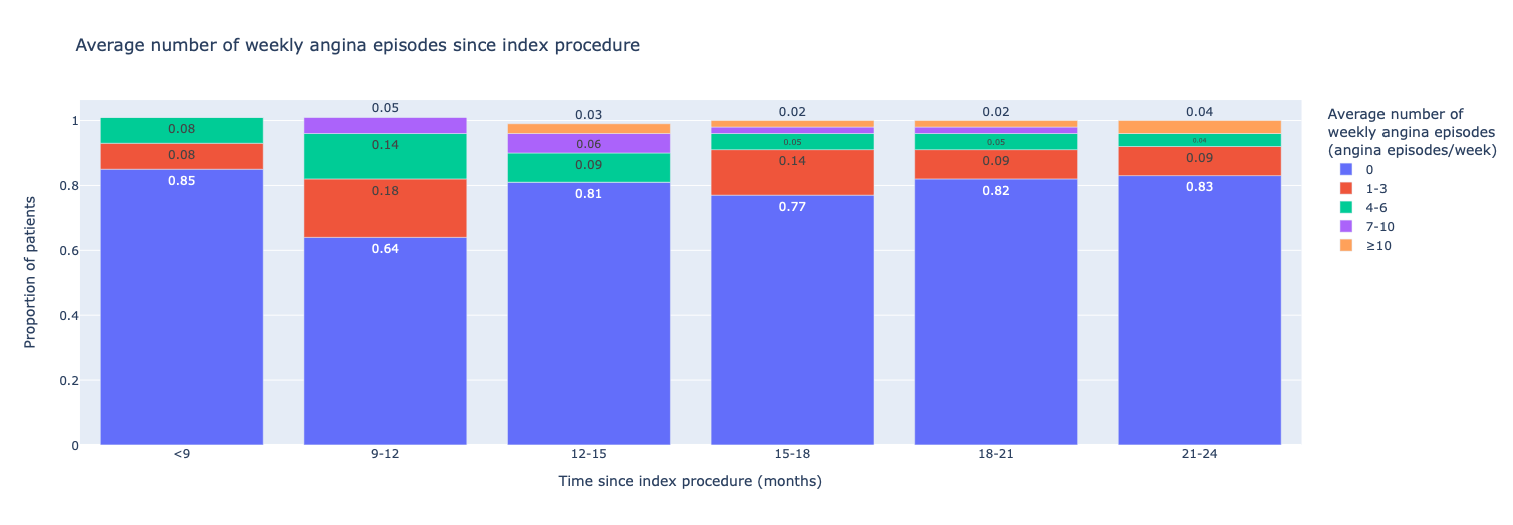


**Number of patients who filled the survey**

| 13 | 22 | 32 | 41 | 55 | 66 |
| --- | --- | --- | --- | --- | --- |

**Number of patients eligible to fill the survey**

| 16 | 29 | 41 | 60 | 76 | 91 |
| --- | --- | --- | --- | --- | --- |


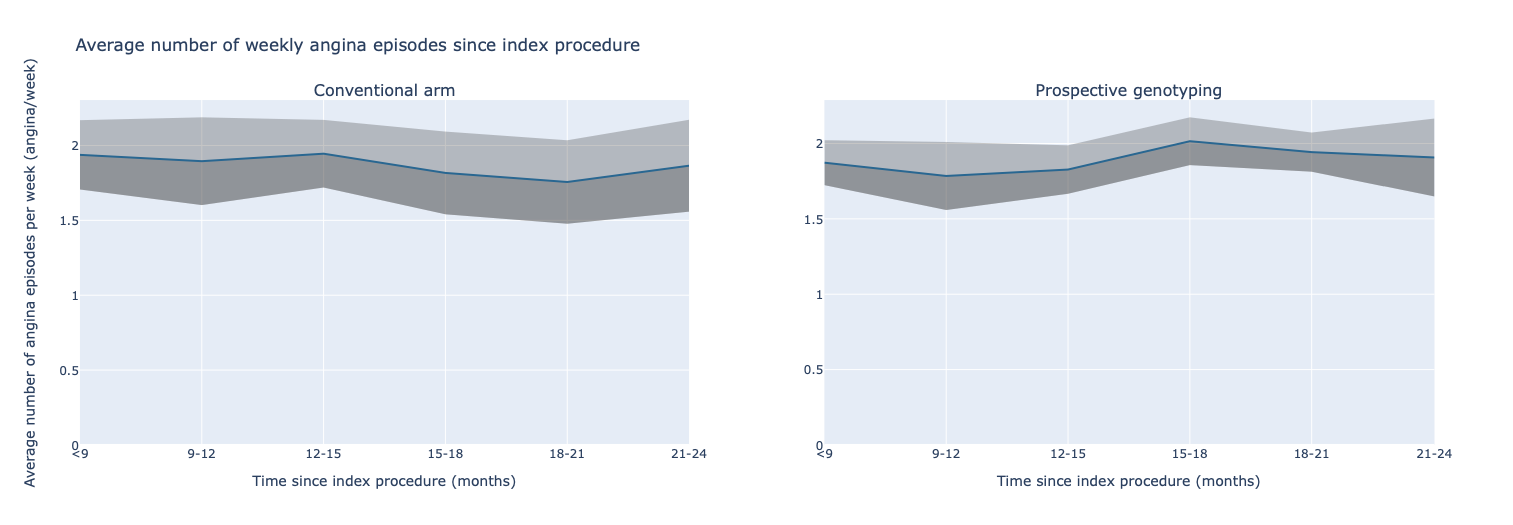


#### B. Shortness of Breath Questionnaire


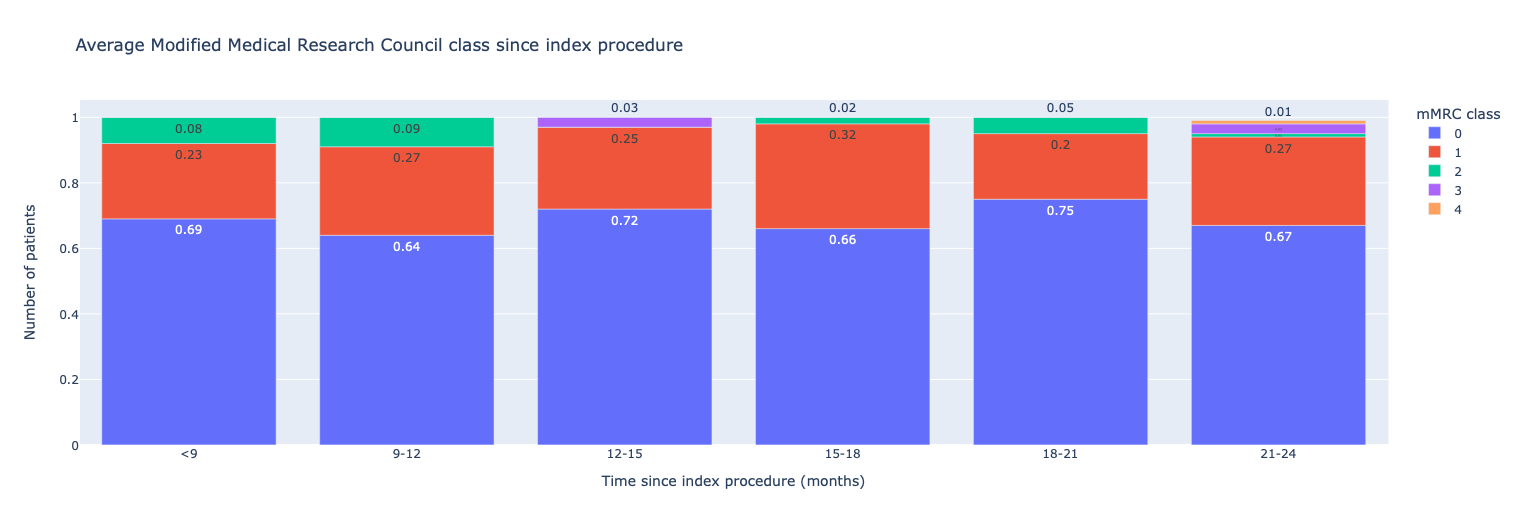


**Number of patients who filled the survey**

| 13 | 22 | 32 | 41 | 55 | 66 |
| --- | --- | --- | --- | --- | --- |

**Number of patients eligible to fill the survey**

| 16 | 29 | 41 | 60 | 76 | 91 |
| --- | --- | --- | --- | --- | --- |


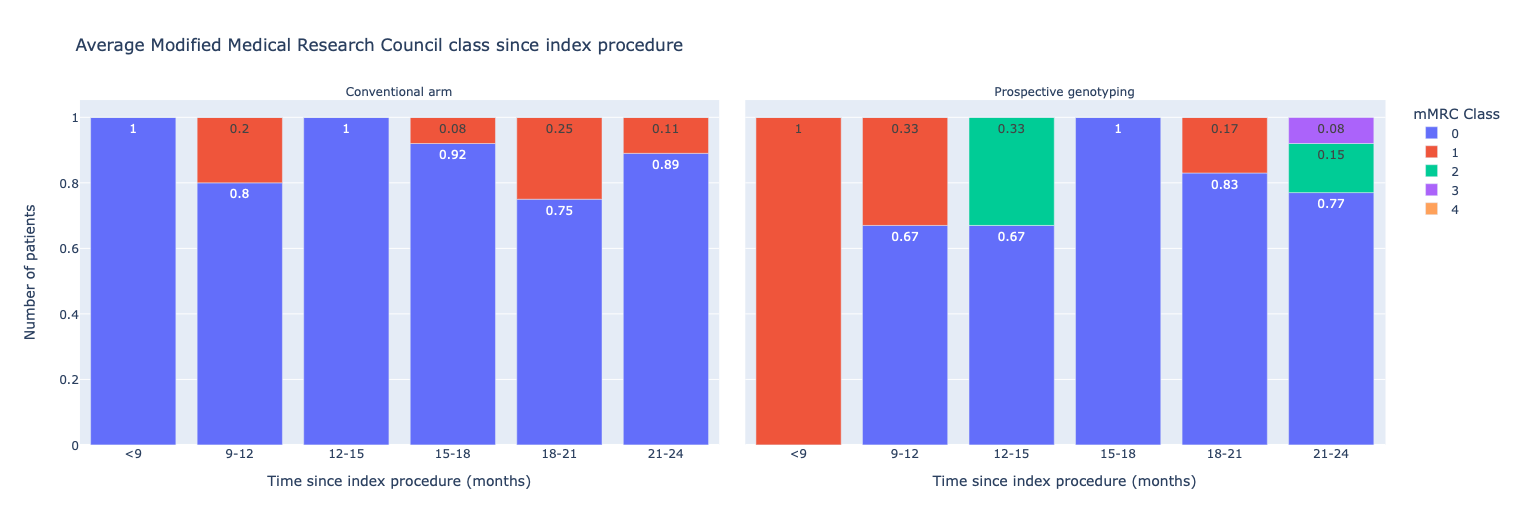


#### C. Seattle Angina Questionnaire


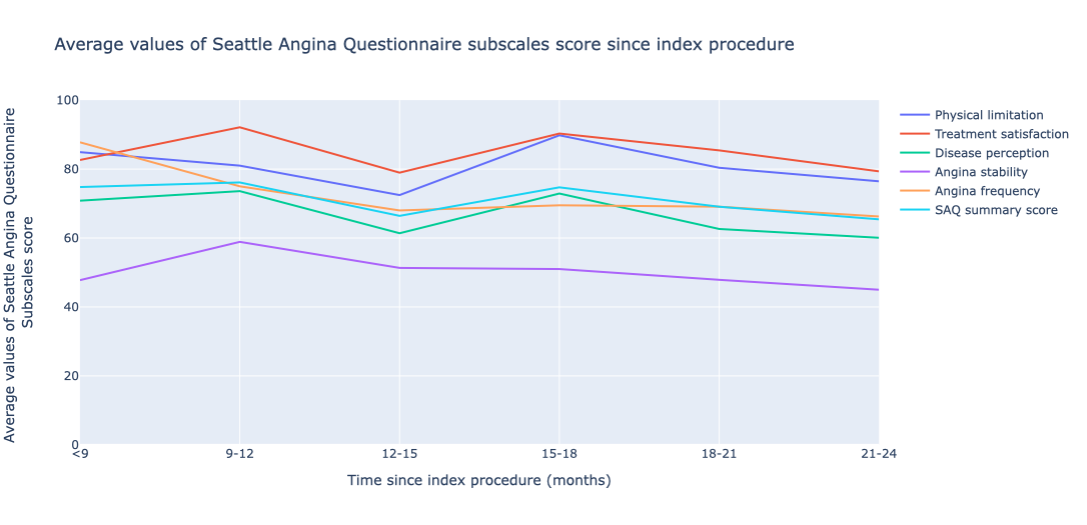


**Number of patients who filled the survey**

| 13 | 22 | 32 | 41 | 55 | 66 |
| --- | --- | --- | --- | --- | --- |

**Number of patients eligible to fill the survey**

| 16 | 29 | 41 | 60 | 76 | 91 |
| --- | --- | --- | --- | --- | --- |

**
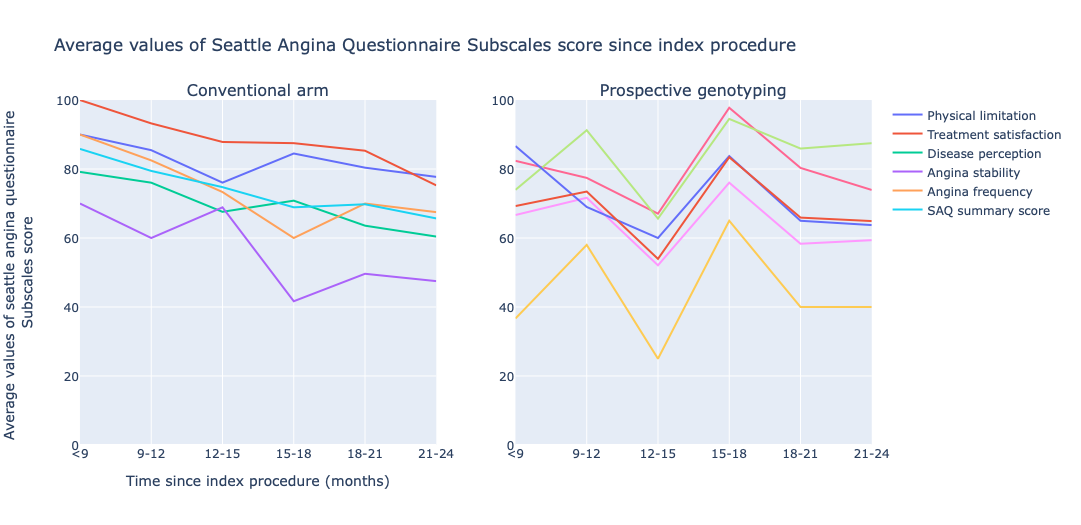
**

#### D. Duke Activity Status Index


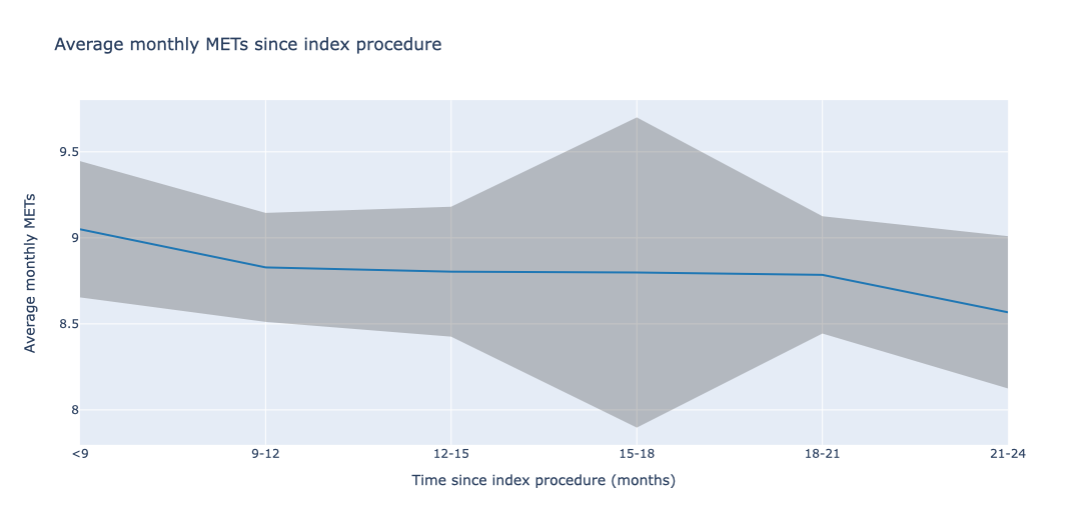


**Number of patients who filled the survey**

| 13 | 22 | 31 | 41 | 53 | 65 |
| --- | --- | --- | --- | --- | --- |

**Number of patients eligible to fill the survey**

| 16 | 29 | 41 | 60 | 76 | 91 |
| --- | --- | --- | --- | --- | --- |


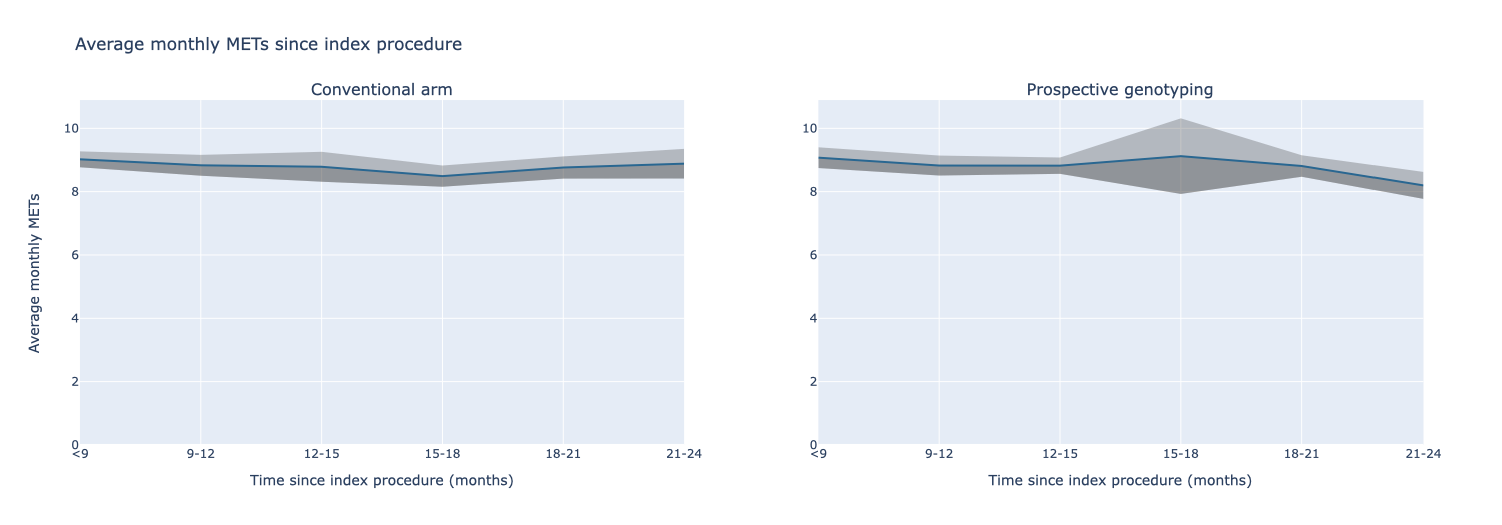


#### E. General Anxiety Disorder-7 Questionnaire


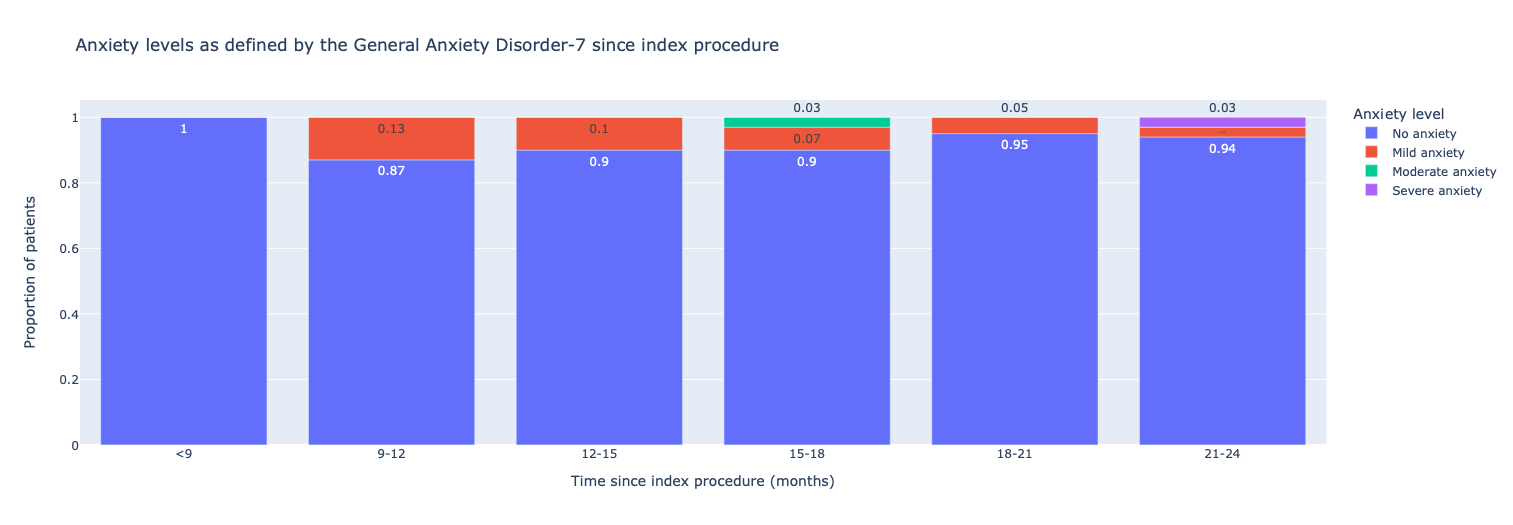


**Number of patients who filled the survey**

| 13 | 15 | 21 | 29 | 37 | 32 |
| --- | --- | --- | --- | --- | --- |

**Number of patients eligible to fill the survey**

| 16 | 29 | 41 | 60 | 76 | 91 |
| --- | --- | --- | --- | --- | --- |

**
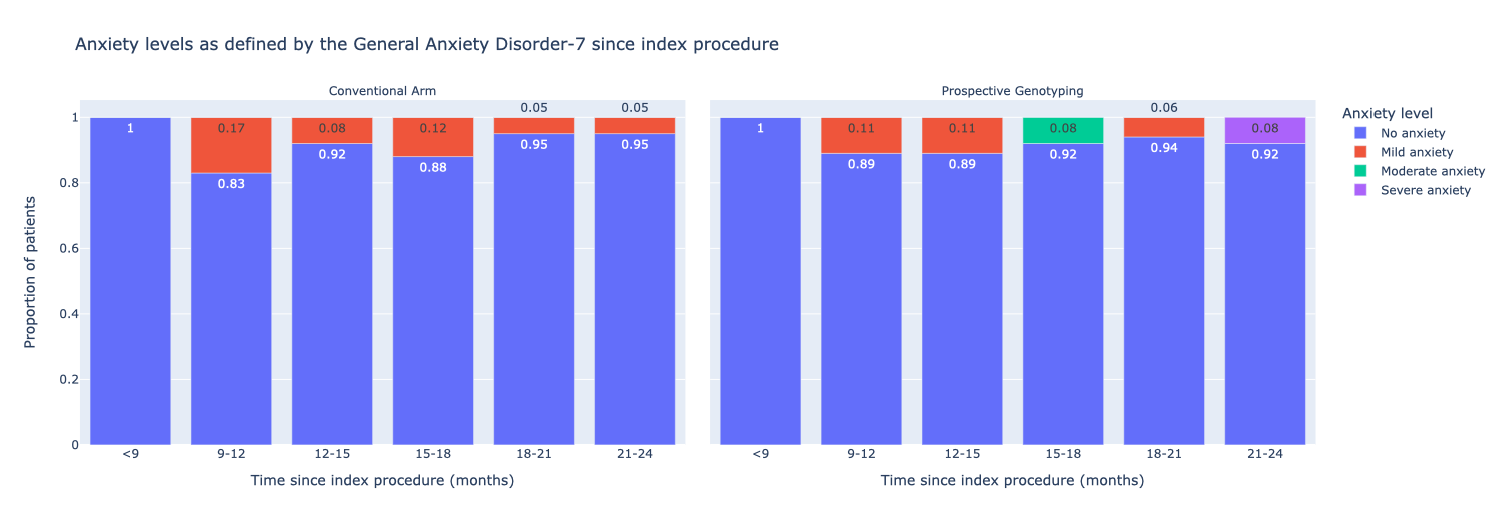
**

**Table S1.** **Multivariable Predictors of Digital Registry Participation**

|  | **Multivariate OR (95% CI)** | | **p-value** |
| --- | --- | --- | --- |
|  | **c-statistic: 0.81** | | |
| **N=907** | |  | |
| **Age and gender interaction** |  | | 0.034 |
| **Age effect within gender** |  | |  |
| Every 5 years of age (women) | 0.89 (0.69-1.16) | |  |
| Every 5 years of age (men) | 1.22 (1.08-1.37) | |  |
| **Gender effect by age** |  | |  |
| Women vs men (age 50) | 1.55 (0.58-4.18) | |  |
| Women vs men (age 55) | 1.14 (0.53-2.47) | |  |
| Women vs men (age 60) | 0.84 (0.46-1.55) | |  |
| Women vs men (age 65) | 0.62 (0.36-1.08) | |  |
| Women vs men (age 70) | 0.46 (0.24-0.87) | |  |
| Women vs men (age 75) | 0.34 (0.15-0.76) | |  |
| Women vs men (age 80) | 0.25 (0.09-0.70) | |  |
| **Diabetes** | 0.55 (0.33-0.95) | | 0.03 |
| **Smoking (Current/recent)** | 0.33 (0.16-0.71) | | 0.004 |
| **Internet use** |  | |  |
| Less than daily | Ref. | | - |
| Daily | 3.72 (1.70-8.20) | | 0.001 |
| Prefer not to answer | 0.19 (0.04-1.06) | | 0.06 |
| **Education** |  | |  |
| <HS/HS grad/some college | Ref. | | - |
| Associate/Bachelor/Graduate/PhD | 2.30 (1.40-3.80) | | 0.001 |
| Prefer not to answer | 2.95 (0.74-11.8) | | 0.13 |
| **Smart speaker** |  | |  |
| No smart speaker | Ref. | | - |
| Has a smart speaker | 1.53 (0.87-2.71) | | 0.14 |
| Prefer not to answer | 1.00 (0.56-1.80) | | 0.99 |

**Abbreviations**: CI: Confidence Interval; Grad: Graduate; HS: High School; LogReg: logistic regression; OR: diagnostic odds-ratio; SD: standard deviation; †p-value is calculated using the Wald test for the odds ratio (two-sided).

#### **Table S2. Questionnaire Results**

|  | **Time Since Index Procedure**  **(months)** | | | | | | |  | |
| --- | --- | --- | --- | --- | --- | --- | --- | --- | --- |
|  | **< 9** | | **9-12** | **12-15** | **15-18** | **18-21** | **21-24** | **p-value *** | |
|  | Questionnaires answers | | | | | | |  | |
| **Eligible to Complete the Questionnaires** | 16 | | 29 | 41 | 60 | 76 | 91 | - | |
| **Duke Activity Status Index** | 9.05±0.40 | | 8.83±0.32 | 8.80±0.38 | 8.80±0.90 | 8.78±0.34 | 8.57±0.44 | 0.004 | |
| **Average mMRC Class** | 0.38±0.65 | | 0.45±0.67 | 0.34±0.65 | 0.37±0.54 | 0.31±0.57 | 0.45±0.80 | 0.16 | |
| **Presence of Angina (% of eligible)** | 3 (18.8%) | | 8 (27.5%) | 5 (12.2%) | 8 (13.35) | 11 (14.4%) | 12 (13.2%) | - | |
| **Average Number of Weekly Angina Episodes** | 1.90±0.18 | | 1.83±0.26 | 1.89±0.19 | 1.91±0.23 | 1.84±0.23 | 1.89±0.28 | 0.09 | |
| **SAQ Physical Limitation** | 84.9±3.1 | | 81.0±4.0 | 72.5±1.6 | 89.8±6.7 | 80.4±1.5 | 76.5±1.43 | 0.06 | |
| **SAQ Angina Frequency** | 87.8±8.2 | | 75.0±4.1 | 68.0±8.7 | 69.5±6.8 | 69.1±6.6 | 66.3±25.0 | 0.36 | |
| **SAQ Angina Stability** | 47.7±11.6 | | 58.9±0.1 | 51.3±11.4 | 51.0±17.8 | 47.9±19.8 | 45.0±0.1 | 0.48 | |
| **SAQ Treatment Satisfaction** | 82.6±9.2 | | 92.1±0.1 | 79.0±13.4 | 90.3±2.2 | 85.4±5.0 | 79.3±0.1 | 0.21 | |
| **SAQ Disease Perception** | 70.8±11.4 | | 73.7±6.9 | 61.4±3.7 | 72.9±2.9 | 62.6±5.3 | 60.1±12.5 | 0.03 | |
| **Overall SAQ Score** | 74.8±11.1 | | 76.1±3.4 | 66.4±4.7 | 74.7±4.3 | 69.1±5.2 | 65.4±9.7 | 0.11 | |
| **GAD7 Score** | 1.2±1.4 | | 2.1±2.7 | 1.4±2.3 | 2.2±3.1 | 1.8±1.9 | 1.9±3.1 | 0.09 | |
| **Antiplatelet therapy** | | | | | | | |  | |
| **Number of patients who last contributed data** | 1 | | 1 | 2 | 8 | 10 | 47 | - | |
| **Aspirin** | 1  (100%) | | 0 (0%) | 1 (50.0%) | 5 (62.5%) | 6 (60.0%) | 28 (59.6%) | - | |
| - With LOF *CYP2C19* | 1  (100%) | | 0 (0%) | 1  (50.0%) | 2 (25.0%) | 1 (10.0%) | 11 (23.4%) | - | |
| - Without LOF - *CYP2C19* | 0  (0%) | 0 (0%) | | 0 (0%) | 3 (37.5%) | 5 (50.0%) | 17 (36.1%) | - | |
| **Clopidogrel** | 0 (0%) | 1 (100%) | | 0 (0%) | 2 (25%) | 7 (70.0%) | 16 (34.0%) | | - |
| - With LOF *CYP2C19* | 0 (0%) | 0 (0%) | | 0 (0%) | 1 (12.5.%) | 1 (10.0%) | 7 (14.9%) | | - |
| - Without LOF   *CYP2C19* | 0 (0%) | 1 (100%) | | 0 (0%) | 1 (12.5%) | 6 (60.0%) | 9 (14.8%) | | - |
| **Ticagrelor** | 0 (0%) | 0 (0%) | | 1 (50.0%) | 1 (12.5%) | 1 (10.0%) | 5 (10.6%) | | - |
| With LOF *CYP2C19* | 0 (0%) | 0 (0%) | | 1 (50.0%) | 1 (12.5%) | 0 (0%) | 4 (8.5%) | | - |
| - Without LOF   *CYP2C19* | 0 (0%) | 0 (0%) | | 0 (0%) | 0 (0%) | 1 (10.0%) | 1 (2.1%) | | - |
| **Abbreviations**: LOF: Loss of Function; mMRC: Modified Medical Research Council; SAQ: Seattle Angina Questionnaire; GAD: General Anxiety Disorder.  *p-value derived using mixed effects models. | | | | | | | | | |

**Additional Material – Telephone Script for Education & Computer Literacy**

Protocol Title: TAILOR-PCI

IRB/REB #:

Principal Investigator:

**Other CRF to have available in preparation of the phone call:**

- Education & Computer Literacy

**Introduction & Reason for Call:**

**Note: The initial introduction and reason for call should be completed using the script for the call at the specified time point. If the patient had already provided written consent for the calls in the second year post PCI and did not give verbal consent for additional questions during this phone call, ask for verbal consent to continue, using the following script. This set of questions and corresponding CRF only needs to be completed one time, in conjunction with any scheduled call.**

*Now I have some questions to ask about your education and computer literacy.*

*There are no known risks from taking part in this research study and you may refuse to answer any question(s) that you do not wish to answer. Please understand your participation is voluntary and you have the right to withdraw your consent or discontinue participation at any time without penalty. Specifically, your current or future medical care at (this medical center) and Mayo Clinic will not be jeopardized if you choose not to participate.*

*Would you be willing to answer some questions to ask about your education and computer literacy?*

**IF YES***:* Continue to Question 1.

**If NO:** *Thank you for your consideration.*

1. *What is the highest degree or level of education you have completed?*

- Less than high school
- High school graduate (or equivalent)
- Some college, no degree
- Associate’s degree
- Bachelor’s degree
- Graduate or professional degree
- Ph.D.
- **(DO NOT READ)** Prefer not to answer

1. *Do you use the internet, and if so, about how often?*

- No
- Yes, about daily
- Yes, about once a week
- Yes, but only occasionally (less than once a week on average)
- **(DO NOT READ)** Don’t know
- **(DO NOT READ)** Prefer not to answer

**Continue as follows, unless patient indicated s/he does not use the internet.**

1. *As I read the following list of items, please tell me if you happen to have each one or not. Do you have…?* Check all that apply.

- A desktop or laptop or netbook computer
- A smartphone (Android, iPhone, Blackberry, etc. which has internet access)
- A tablet computer
- A smart-speaker (Alexa, Google Home or Apple HomePod or other)
- A regular cell phone (e.g. a cell phone without internet access)
- **(DO NOT READ)** Prefer not to answer

1. *From the list of previous items that you have, which one do you use the most to access the internet?*

- A desktop or laptop or netbook computer
- A smartphone (Android, iPhone, Blackberry, etc. which has internet access)
- A tablet computer
- A smart-speaker (Alexa, Google Home or Apple HomePod or other)
- **(DO NOT READ)** Prefer not to answer

**If the patient indicated s/he uses has a smartphone in Question 3, continue as follows.**

1. Have you ever downloaded a software application or “app” to your cell phone?

- Yes, patient has done this
- No, patient has never done this
- Phone cannot download apps
- **(DO NOT READ)** Don’t know
- **(DO NOT READ)** Prefer not to answer

*Thank you for answering these questions. Please understand that your answers will remain confidential.*
